# Reinvestigating the status of malaria parasite (*Plasmodium* sp.) in Indian non-human primates

**DOI:** 10.1371/journal.pntd.0006801

**Published:** 2018-12-06

**Authors:** Jyotsana Dixit, Arun Zachariah, Sajesh P. K., Bathrachalam Chandramohan, Vinoth Shanmuganatham, K. Praveen Karanth

**Affiliations:** 1 TE-11, Centre for Ecological Sciences, Indian Institute of Sciences, Bangalore, India; 2 Department of Forests and Wildlife, Sulthan Batheri, Wayanad District, Kerala State, India; 3 Scigenom Research Foundation, Cochin, Kerala, India; 4 School of Biological Sciences, National Institute of Science Education and Research, Bhubaneshwar, Odisha, India; Universidad Peruana Cayetano Heredia, PERU

## Abstract

Many human parasites and pathogens have closely related counterparts among non-human primates. For example, non-human primates harbour several species of malaria causing parasites of the genus *Plasmodium*. Studies suggest that for a better understanding of the origin and evolution of human malaria parasites it is important to know the diversity and evolutionary relationships of these parasites in non-human primates. Much work has been undertaken on malaria parasites in wild great Apes of Africa as well as wild monkeys of Southeast Asia however studies are lacking from South Asia, particularly India. India is one of the major malaria prone regions in the world and exhibits high primate diversity which in turn provides ideal setting for both zoonoses and anthropozoonoses. In this study we report the molecular data for malaria parasites from wild populations of Indian non-human primates. We surveyed 349 fecal samples from five different Indian non-human primates, while 94 blood and tissue samples from one of the Indian non-human primate species (*Macaca radiata*) and one blood sample from *M*. *mulatta*. Our results confirm the presence of *P*. *fragile*, *P*. *inui* and *P*. *cynomolgi* in *Macaca radiata*. Additionally, we report for the first time the presence of human malarial parasite, *P*. *falciparum*, in *M*. *mulatta* and *M*. *radiata*. Additionally, our results indicate that *M*. *radiata* does not exhibit population structure probably due to human mediated translocation of problem monkeys. Human mediated transport of macaques adds an additional level of complexity to tacking malaria in human. This issue has implications for both the spread of primate as well as human specific malarias.

## Author summary

Non-human primates are our closest relatives and with them we share many of our disease-causing pathogens. Malaria is one such example where the parasite (*Plasmodium*) causing malaria in humans has originated from non-human primate populations. To understand origin and evolution of human malaria parasites it is important to know the diversity and evolutionary relationships of these parasites in non-human primates. In this study we report the molecular data for malaria parasites from wild populations of Indian non-human primates. We surveyed 349 fecal samples from five different Indian non-human primates, while 94 blood and tissue samples from one of the Indian non-human primate species (*Macaca radiata*). Our results confirm the presence of *P*. *fragile*, *P*. *inui* and *P*. *cynomolgi* in *Macaca radiata*. Additionally, we report for the first time the presence of human malarial parasite, *P*. *falciparum*, in *M*. *mulatta* and *M*. *radiata*.

## Introduction

In the last two decades much work has been done to understand the evolutionary origin of human malarial parasite. Molecular phylogeny of *Plasmodium* infecting primates suggests that the principal malaria causing pathogens in humans (*P*. *falciparum*, *P*. *vivax*, *P*. *malariae*, and *P*. *ovale*) are related to *Plasmodium* infecting non-human primates and have multiple, independent evolutionary origins [1–4]. For example, studies indicate that *P*. *falciparum* is of gorilla origin and not of ancient human origin [5]. The largely Asian malarial parasite *P*. *vivax* also appears to have an African origin as it is related to *Plasmodium* infecting the great apes of Africa [6]. These studies indicate that to understand the origin, evolution and transmission of these primate-derived human pathogens it is imperative that we understand the diversity and phylogenetic affinity of these pathogens in their natural hosts, the non-human primates (henceforth referred to as primates). Additionally, continuous monitoring of *Plasmodium* diversity in wild primates can alert us to new *Plasmodium* species that might spread to humans. For example, the recently detected *knowlesi* malaria in human from Southeast Asia was acquired from wild macaques which serve as their reservoir hosts [7–11].

Studies have also shown that primates serve as reservoirs for *Plasmodium* recently acquired by humans from primates [12–14]. For example, *P*. *falciparum*-related pathogens can naturally circulate in some monkey populations in Africa [15, 16]. Additionally, now there is evidence of multiple transfer of *Plasmodium* from human to primates [4, 15–20]. This is because, even if malaria is completely eradicated from humans, in the long run animal reservoirs can provide source for recurrent malaria infection [21–23]. Such anthropozoonoses (pathogens transmitted from humans to animal populations) also have important conservation implication given many primate species are threatened or endangered and their populations might decline due to spread of human malaria to primates.

With over 15 species of primates, India is among the countries with high primate diversity [24]. Out of these 15 species three are widely distributed while the remaining species have restricted distributions. The widely distributed species include, Hanuman langur (*Semnopithecus sp*.) distributed all over India, rhesus macaque (*Macaca mulatta*) distributed in north India, and bonnet macaque (*Macaca radiata*) distributed in South India. These monkeys are quite common across India and also found in cities and villages. Additionally, they are considered sacred and are often provisioned by humans. While in other parts of India these monkeys’ raid crops and are considered as pests [25]. Thus, there is much interaction between humans and primates in India which in turn provides ample opportunity for disease transmission. However, very little is known about prevalence, diversity and spread of malarial parasites in Indian primates. Till date at least three primate specific *Plasmodium* species have been reported from *M*. *radiata* in South India, these include *P*. *inui*, *P*. *cynomolgi* and *P*. *fragile*[26–29]. In Sri Lanka these parasites have been reported from *M*. *sinica* which is closely related to *M*. *radiata*. Additionally, *P*. *cynomolgi* has also been reported from langurs (*Semnopithecus priam*) in Sri Lanka [30, 31]. Both *P*. *inui* and *P*. *cynomolgi* also infect many Southeast Asian primates, whereas *P*. *fragile* is endemic to the macaques of India and Sri Lanka. Interestingly, most of these reports of malaria in wild Indian primates are from the high rainfall regions of Southwest India probably due to the restricted distribution of the vector, *Anopheles elegans*, which is confined to evergreen forests [32–34].

Given that India is one of the major malaria prone regions in the world and exhibits high primate species richness, it is plausible that human *Plasmodium* (particularly *P*. *falciparum* and *P*. *vivax*) might have been transferred to Indian primates and these primate populations might also represent a source for recurring human malarial infection. Consequently, Indian primates might represent both source and sink of *Plasmodium* infecting humans. Furthermore, Indian primates may harbour many more *Plasmodium* species than that has been reported thus far. For example, genetic studies suggest that there exist numerous “cryptic” species of *Plasmodium* and other genera of malarial parasites in lizards and birds, hence the current estimates of the diversity of malaria causing protists are likely to be low [1]. The close interaction between humans and primates in India might facilitate both zoonoses and anthropozoonoses. Additionally, the unregulated human mediated translocation of “problem monkeys” might further hasten the spread of these pathogens. Till date there has been no published report on *Plasmodium* genetic diversity and their phylogenetic affinity in free ranging Indian primates. However, such studies have been undertaken on Southeast Asian (SEA) primates [7, 35–38]. Thus, here we attempt to address the following questions: What are the different species of *Plasmodium* infecting Indian primates? What are the phylogenetic relationships between these *Plasmodium* species and how are they related to *Plasmodium* species isolated from other primates as well as humans? What effect has human mediated translocation had on the population structure of widely-distributed host species?

To address these questions, we collected liver and spleen tissues, blood and fecal samples from multiple primate species from India. Much of the sample collection was concentrated along Southwest India where simian malaria has been reported previously. These samples were used to amplify both host and parasite markers to better understand host population structure and simian malaria diversity in India.

## Materials and methods

### Samples

#### Fecal samples

**Fig 1:**
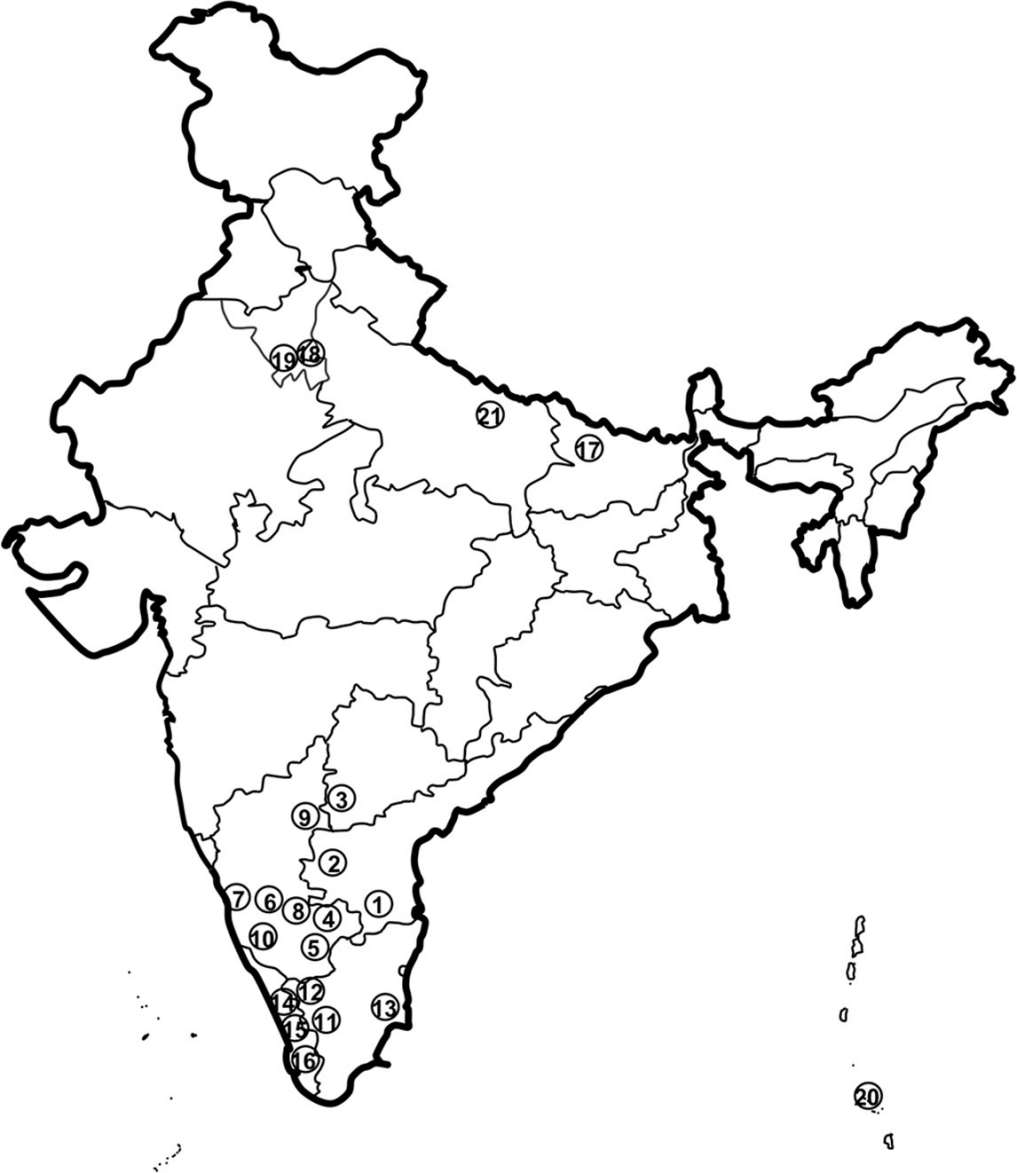
Details of samples collected from different locations of India used for the present study.

Fecal samples from five species of primates (*Macaca radiata*, *M*. *mulatta*, *M*. *sinica*, *M*. *fascicularis umbrosa*, and *Symnopithecus hypoleucus*) were collected during the years 2014–15 across seven states (Karnataka, Kerala, Andhra Pradesh, Telangana, Delhi, Bihar and Andaman and Nicobar Islands) of India. The location details of samples collected from each species of primates are shown in Fig 1 and S1 Table. The whole and sometimes portions of the freshly collected fecal material were stored in sterile vials in 70% alcohol or a 1:1ratio with RNA later at room temperature in field conditions and were stored in laboratory at -20°C until DNA extraction.

#### Blood and tissue samples

In the case of *Macaca radiata* from Kerala and few from Karnataka blood and tissue samples were provided by other researchers. Twenty seven samples from Waynad (11 liver, 8 spleen and 8 blood) and twelve blood samples from Kolpetta, were collected by Kerala forest department from the dead animals found in the forest during the year 2014–15. Fifty-one more blood samples were obtained from captive macaques from Trichur Zoo (Kerala) during 2015. Furthermore during 2015 four blood samples from Karnataka were obtained from Primate Research Centre (PRC) at Indian Institute of Sciences (IISc Bangalore) campus, where the blood from macaques were drawn for routine screening for diseases. Blood sample of a captive *M*. *mulatta* infected with *P*. *cynomolgy* under laboratory condition was obtained from CDRI Lucknow.

### Molecular data fecal samples

#### DNA extraction from fecal samples and host DNA detection

DNA from the fecal samples was extracted by QIAamp fast DNA stool kit (Qiagen). Each fecal sample was then tested for the presence of the host DNA by using published set of primers specific for each host species. For *M*. *radiata* we used published set of primers LqqF: 5’ TCCTAGGGCAATCAGAAAGAAAG and TDKD: 5’ CCTGAAGTAGGAACCAGATG, for amplifying the ~540 bp of mitochondrial D-loop region [39]. For *M*. *fascicularis umbrosa*, and *M*. *mulatta* we used another published set of primers Saru-4F: 5’ ATCACGGGTCTATCACCCTA and Saru-5r: 5’ GGCCAGGACCAAGCCTATTT for amplifying 630 bp of mitochondrial D-loop region [40]. For *M*. *Silenus* we utilized the published set of primers D1: 5’ GTACACTGGCCTTGTAAACC 3’) and D3: 5’ CTTATTTAAGGGGAACGTGTGG 3’, for hypervariable region-I (HVR-I) to detect the host DNA (amplicon size 650 bp)[41]. For detecting the presence of *Semnopithecus hypoleucos* DNA in the fecal material the mitochondrial Cytochrome *b* (*Cyt-b*) region was amplified using primers Langur_CytbF: 5’ ATTATCGCARCCTTCACAATC and L2_CytbR: 5’ TTGTGRAGTATRGGTAYRATTGTC (amplicon size 320 bp)[42]. For all the primer pairs the published annealing temperature and PCR cycle conditions were followed. A total of 4μl of the PCR product were visualized by gel electrophoresis on a 2% agarose gel. The samples showing amplification for the host DNA were then subjected to malaria parasite screening and further analysis, while the samples showing no amplification were not included in further analysis.

#### Malaria diagnostic PCR using cytochrome b gene from fecal samples

Every fecal sample found positive for the host DNA was then screened for the presence of malaria parasite using primers specifically designed using the PrimerSelect computer program (a component of the DNASTAR, Madison, WI, USA) to amplify 200bp fragment of *Plasmodium* Cytochrome *b* (*Cyt-b*) gene. The *Cyt-b* external primers *Cyt-b*3F: 5’GGWCAAATGAGTTATTGRG and *Cyt-b* 3R: 5’CATAGAATGMACACATAAACC amplified a 350-bp PCR product and internal primers *Cyt-b* 2F: 5’GGTAGCACWAATCCYTTAGGG and *Cyt-b* 2R: 5’GGTARAAARTACCATTCWGG amplified 200-bp region of the parasite *Cyt-b* gene. The primary PCR amplification using the external primers was carried out in a 25μl volume reaction using 2 μl of extracted total genomic DNA, 1.5mM MgCl_2_, 1x PCR buffer, 0.25mM of each deoxynucleoside triphosphate, 0.4mM of each external primer and 0.25 U/μl of NEB taq Polymerase (New England Biolabs Inc.). The primary PCR conditions were: initial denaturation at 95°C for 5 min, followed by 35 cycles (94°C for 40 secs, 45°C for 30 sec and 72°C for 30 sec) and final extension at 72°C for 4 min. Second nested PCR was of 25 μl total volume and consisted of2 μl of the primary PCR product, 0.3 μl (1.5 Unit) of Taq DNA Polymerase (New England Biolabs Inc.), 1mM dNTPs, 1 μM of each internal primer. The cycling conditions were same as above except the number of cycles were restricted to 25. The fecal sample from laboratory infected (*P*. *cynomolgy*) *M*. *mulatta* was utilized as a positive control for the PCRs.

### Molecular data blood and tissue samples

#### DNA extraction from blood and tissue samples and malaria diagnostics

DNA from blood and tissue samples was extracted using DNeasy blood and tissue kit (Qiagen, Germany) according to the standard protocol. Each sample was then screened for the presence of malaria parasites by nested PCR, using primers for a 1,200 bp fragment of *Cyt-b* gene that have been used in previous studies [36] and the references therein. The *Cyt-b* external primers were forward: 5’TGTAATGCCTAGACGTATTCC and reverse: 5’GTCAAWCAAACATGAATATAGAC and the internal primers were Forward: 5’ TCTATTAATTTAGYWAAAGCAC and Reverse: 5’GCTTGGGAGCTGTAATCATAAT. The primary PCR amplifications were carried out in a 25μl volume reaction using 20 ng of total genomic DNA, 1.5mM MgCl_2_, 1xPCR buffer, 0.6mM of each deoxynucleoside triphosphate, 0.4mM of each primer, and 0.25 U/μl NEB Taq polymerase (New England Biolabs). The primary PCR conditions were same as mentioned in [36]. A total of 25μl of the secondary PCR was run using 0.1 μl (0.5Unit) of Taq DNA Polymerase (New England Biolabs Inc.), 0.6mM dNTPs, 0.4 μM of each primer (Forward and Reverse). Secondary PCR thermal cycling conditions were the same as mentioned in [36]. The blood sample from laboratory infected (*P*. *cynomolgy*) *M*. *mulatta* was utilized as a positive control for the PCRs.

The 4ul of *Cyt-b* gene amplified products were visualized through electrophoresis on 2% agarose gel and successfully amplified products were subjected to direct sequencing using ABI 3730 capillary sequencer and identified as *Plasmodium* using BLAST [43].

#### Nuclear marker data from parasite

Apart from mitochondrial marker, we further attempted to amplify portions of two nuclear genes (1) *MSP-1*_*42*_ (encoding a major antigen in the parasite) and (2) *18s rRNA* from blood and tissue samples positive for *Plasmodium*.

1. Previously published primer pairs were used to amplify ~900 bp region of *MSP-1*_*42*_ gene Forward: 5’GACCAAGTAACAACGGGAG and Reverse: 5’CAAAGAGTGGCTCAGAACC [36], the PCR was done in a final volume of 25μl with 20 ng of total genomic DNA, 1.5mM MgCl_2_, 1xPCR buffer, 0.6mM of each deoxynucleoside triphosphate, 0.4mM of each primer, and 0.03 U/μlAmpliTaq Gold DNA polymerase (Applied Biosystems, Roche-USA). PCR thermal cycling conditions were the same as mentioned in [36].
2. For *18s rRNA* gene nested PCR was conducted using two sets of primers. For primary PCR we used the published primer pair rPLU1: 5’ TCAAAGATTAAGCCATGCAAGTGA and rPLU5: 5′ CCTGTTGTTGCCTTAAACTCC [44]. For the secondary PCR the presently designed primers were used rUNIF1: 5' TTAAGCCATGCAAGTGAAAGTAT-3' and rUNIR1: 5'-CGGTATCTGATCGTCTTC. The first PCR amplification was carried out in a final volume of 25μl, which included 10pmol of each primer pair, 0.2mM of dNTP, 1 U taq Polymerase (NEB Biolabs), 1x PCR buffer and 20ng of the total genomic DNA. Cycling conditions included an initial denaturation at 95°C for 5 min followed by 35 cycles of 1 min denaturation at 95°C, 1 min annealing at 55°C, 1 min of extension at 72°C followed by 5 min final extension at 72°C. 4μl of the primary PCR product was then used as template for the secondary PCR with a final volume of 25μl which included 10pmol of each primer pair, 0.2mM of dNTP, 1 U taq Polymerase (NEB Biolabs) and 1x PCR buffer.

Approximately 4μl of PCR product of each amplified DNA fragment for both the genes (*18s rRNA*and*MSP-1*_*42*_gene) was electrophoresed on a 2% agarose gel, utilizing a 100-bp ladder (BangloreGenei) to confirm amplicon size. Successfully amplified products were purified by incubation with Exonuclease-I and Shrimp Alkaline Phosphatase (Fermentas, Life Sciences) in a thermal cycler at 37°C for 120 min, followed by enzyme inactivation at 85°C for 15 min and subjected to sequencing from both the ends and identified using BLAST search.

### Phylogenetic analysis using parasite molecular data

Parasite nuclear and mitochondrial markers sequenced from host *M*. *radiata* blood and tissue samples were subjected to phylogenetic analyses. Separate alignments of one mitochondrial (*Cyt-b*) and two nuclear (*MSP-1*_*42*_, and *18s rRNA*) markers were generated using ClustalW and Muscle as implemented in MEGA 5.2 [45]with manual editing. We used GTR+I+G model for all phylogenetic analyses based on results from JModelTest 2.1.2 [46]. Phylogenetic relationships were estimated using Maximum likelihood and Bayesian methods using RAxML and MrBayes respectively. Bayesian tree was constructed in MrBayes [47]with the following settings: Markov Chain was run for 4x10^6^ generations where sampling was performed every 100 generations, the chains were assumed to have converged once the average standard deviation of posterior probability was below 0.01, first 25% of the trees were discarded as burn-in. The ML tree was generated using the program RAxML GUI [48] with 500 bootstrap replicates using rapid bootstrap settings. S1–S3 Tables provide a complete list of the published sequences utilized for the present study phylogenetic analysis. The African primate parasite *P*. *gonderi* was used to root the phylogeny for *Cyt-b*and *18s rRNA* gene trees, given that previous studies have shown that SEA simian parasites originated in Africa [2, 49], while for *MSP-1*_*42*_ gene *P*. *fragile* sequence was used to root the tree as used in previous study [36].

### Host molecular data using fecal samples

Human mediated translocations of host species can significantly alter the distribution of their parasites. To better understand host population structure, we sequenced mitochondrial D-loop region from 82 samples of *M*. *radiata* (which showed both the presence and absence of parasite) representing all the states where they are distributed. The PCR components and cycling conditions were same as mentioned above and in [39].

#### Host phylogenetic analysis

The D-loop sequences of the host (*M*. *radiata*) were aligned using Muscle in MEGA 5.2. Based on JModelTest 2.1.2, we used a time reversible model with gamma-distributed substitution rates and a proportion of invariant sites (GTR+G+I) for constructing phylogenetic relationships among *M*. *radiata* populations. The phylogenetic inferences were made using Maximum likelihood and Bayesian methods as implemented in RAxML and MrBayes respectively using settings described above.

#### Host haplotype network and IBD model

A minimum spanning network for a complete set of 82 *M*. *radiata* mtDNA sequences was estimated using PopART [50] with epsilon parameter set to 0. Further to test whether *M*. *radiata* mtDNA data fit the isolation-by-distance (IBD) model of population structure [51] the programme IBD v1.5 [52] was used to determine the correlation between pairwise genetic distances (p distance) and the geographic distances between population pairs.

## Results

### Fecal samples

#### Host identification and sequencing of host mitochondrial genes

A total of 349 fecal samples from five different Indian primate species were collected and tested for the presence of host DNA by sequencing mitochondrial regions (details as given in Table 1 and S1 Table). Maximum number of fecal samples were collected from *M*. *radiata* where out of 251fecal samples collected 120 were found positive for the presence of host DNA. Fifteen fecal samples out of 24 samples collected for *M*. *mulatta* were found positive for host DNA. For *M*. *fascicularis umbrosa* a total 57 fecal samples were collected and 30 were found positive for host DNA, while for *Symnopithecus hypoleucus* 11 fecal samples were collected from two different locations out of which nine samples were positive for host DNA. Furthermore, four out of six fecal samples collected for *Macaca silenus* were found positive for host DNA. Details of fecal samples collected from each of the species studied and numbers of samples found positive for host DNA are listed in Table 1.

**Table 1:** Details of fecal samples collected, and *Plasmodium* species determined by cytochrome *b* sequences in Indian primates.

S. No.	Host species	Field sites sampled	Fecal samples tested for host DNA	Fecal samples found +ve for host DNA	Number of cytochrome *b* sequences obtained for *P*. *falciparum*
1	*Macaca radiata*	16	251	120	19
2	*Macaca mulatta*	04	24	15	1
3	*Macaca fascicularis umbrosa*	04	57	30	-
4	*Symnopithecus hypoleucus*	02	11	09	-
5	*Macaca silenus*	01	06	04	-

#### Screening host fecal samples for parasite DNA

Total 178 (host DNA positive) fecal samples of five different Indian primate species collected from different locations of India were tested for the presence of *Plasmodium* parasite using nested PCR. Interestingly, except *M*. *radiata* and one sample from *M*. *mulatta* all the fecal samples from other primates were found to be negative for the presence of malaria parasite. Out of total 120 fecal samples tested from *M*. *radiata* 19 samples (16%) showed the presence of parasite however, out of 15 samples from *M*. *mulatta* tested only one showed the presence of parasite (6.6%) (Table 1). With nested PCR a 200 bp fragment was obtained from all the parasite positive samples. Sequence comparison with the published data (based on 200 bp region) showed that all the sequences were identical to *P*. *falciparum Cyt-b* gene sequences. However, we were not able to sequence any bigger fragment of the parasite genome (mitochondrial or nuclear) from these samples, probably due to poor quality of the DNA obtained from fecal samples.

### Blood samples

#### Screening host blood and tissue samples for parasite DNA

A total of 94 blood and tissue samples from *M*. *radiata* collected from four different locations from Southern India were tested for the presence of the *Plasmodium* parasites (Table 2). None of the captive macaques from Trichur zoo were found positive for *Plasmodium*, however in wild samples from Waynad and Kolpetta the prevalence of this parasite was very high (see below). Out of 27 blood and tissue samples collected from Waynad, 12 samples (5 spleen, 6 liver and 1 blood) were found positive for the parasite DNA, while five samples out of 12 blood samples collected from Kolpetta were parasite positive. Furthermore, the parasite was found in two captive *M*. *radiata* macaques from PRC IISc campus (Table 2).

**Table 2:** Details of *Plasmodium* species detected using blood and tissue samples of *M*. *radiata* species.

S. No.	Field sites tested	Samples tested	*Cyt-b* gene sequences			*MSP-1*_*42*_ gene sequences			*18s rRNA* gene sequences		
			Spleen	Liver	Blood	Spleen	Liver	Blood	Spleen	Liver	Blood
1	Waynad (kerala)	27*	*P*. *inui* (4), *P*. *fragile* (1)	*P*. *inui* (2), *P*. *fragile* (2), *P*. *falciparum* (2)	*P*. *inui* (1)	*P*. *inui* (2)	0	0	*P*. *inui* (3)	*P*. *inui* (1)	*P*. *inui* (1)
2	Kolpetta (Kerala)	12*	0	0	*P*. *inui* (3), *P*. *fragile* (2)	0	0	*P*. *inui* (3)		0	*P*. *inui* (2)
3	Trichur (Kerala)	51	0	0	0	0	0	0	0	0	0
4	Primate Research Centre, IISc, Bangalore (Karnataka)	04	0	0	*P*. *inui* (1), *P*. *cynomolgi* (1)	0		0	0	0	*P*. *inui* (1), *P*. *cynomolgi* (1)
5	CDRI, Lucknow	01				0	0	*P*. *cynomolgi* (1)	0	0	0

* wild samples.

#### *Plasmodium* species identification

Mitochondrial *Cyt-b* gene sequenced from positive samples were subjected to a BLAST search to identify the *Plasmodium* species. We were able to amplify and sequence 19 *Plasmodium Cyt-b* gene sequences from Indian *M*. *radiata*. BLAST analysis showed 11 *P*. *inui*, five *P*. *fragile* two *P*. *falciparum* and one *P*. *cynomolgi* sequences (Table 2). A total of 61 sequences (published and presently generated) were aligned using clustal W in MEGA 5.2 (S2 Table) for phylogenetic analysis. The total length of the aligned sequences was 960 positions. Interestingly no variation was seen among Indian *P*. *inui* sequences. However, the divergence between Indian and SEA samples was 0.016 (Table 3). The divergence among Indian and Sri Lankan *P*. *fragile* was 0.005 (Table 3). Interestingly, only a single isolate of *P*. *cynomolgi* was found in the present study, and it’s not very divergent from the rest of the SEA sequences (0.004, Table 3). The *P*. *falciparum* partial *Cyt-b* gene sequence isolated from *M*. *radiata* was identical to *P*. *falciparum* found in humans. To find any difference we further need to sequence the whole mitochondrial genome from these isolates, but we were unable to do so, may be due to very low parasitemia in presently studied macaques.

**Table 3:** Genetic divergences (substitutions per site) among different *Plasmodium s*pecies using the Kimura 2-Parameter model as implemented in MEGA v5.2.2 [45].

Species	Genetic distance		
	*Cyt-b*	*18s rRNA*	*MSP-1*_*42*_
*P*. *inui* (overall mean distance)	0.014 (30)	0.012 (39)	0.029 (22)
*P*. *inui* (Indian clade mean distance)	0 (11)	0 (08)	0.015 (5)
*P*. *inui* (SEA clade mean distance)	0.015 (19)	0.010 (31)	0.026 (17)
*P*. *inui* (Indian clade vs SEA clades)	0.016 (11–19)	0.004 (08–31)	0.036 (5–17)
*P*. *fragile* (Overall mean distance)	0.004 (8)	0.001 (5)	*
*P*. *cynomolgi* (Overall mean distance)	0.006 (18)	0.071 (6)	0.042 (11)
*P*. *cynomolgi* (SEA clade mean distance)	0.006 (19)	0.055 (5)	0.044 (10)
*P*. *cynomolgi* (Indian vs SEA)	0.004 (1–18)	0.10 (1–5)	0.029 (1–10)

* Sequences not available; numbers in bracket are sample size.

#### *Plasmodium* phylogenetic analysis using *Cyt-b* gene sequences

**Fig 2:**
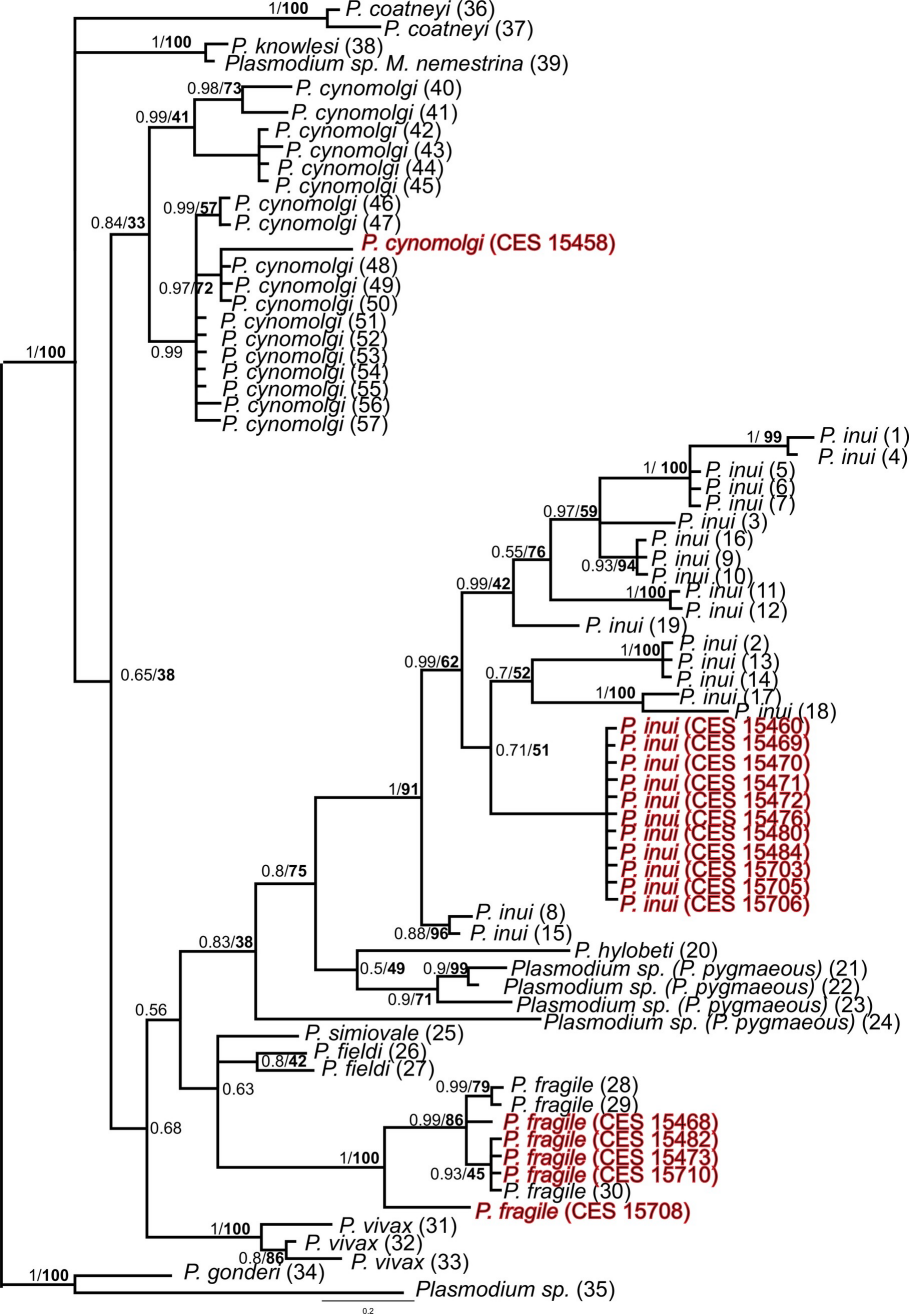
Phylogenetic tree of *Plasmodium* species based on mitochondrial *Cyt-b* gene region. Bayesian and ML methods yielded similar tree topologies and so only Bayesian tree is shown. The values above branches are posterior probabilities together with the bootstrap values (in bold) in percentage obtained for ML tree.

We used Maximum likelihood and Bayesian methods to construct the phylogenetic tree of *Plasmodium* species found in Indian macaques. The overall phylogeny was similar for the two methods, thus only the Bayesian tree is shown in Fig 2.

Overall, this phylogeny is like those obtained in previous studies [36], but the present phylogeny additionally includes *Cyt-b* gene sequences of *P*. *inui*, *P*. *fragile* and *P*. *cynomolgi* found in Indian macaque *M*. *radiata*. The *P*. *inui Cyt-b* gene sequences derived from *M*. *radiata* were monophyletic and this clade was nested within the radiation of *P*. *inui* derived from SEA macaques. In case of *P*. *fragile* the Indian sequences show some variation and together with Sri Lankan sequences form a separate clade. The single *P*. *cynolmolgi* sequence found in this study fall within *P*. *cynomolgi* clad along with sequences from Sri Lanka and SEA. Furthermore, as expected the *Cyt-b* gene sequences from *P*. *falciparum* samples obtained from Indian macaques were identical to the published *P*. *falciparum* sequences. All the newly generated *Plasmodium Cyt-b* gene sequences were deposited in GenBank sequence repository under accession numbers: *P*. *inui* (MH974123-MH974133), *P*. *falciparum* (MH974139-MH914140), *P*. *cynomolgi* (MH974122), *P*. *fragile* (MH974134-MH974138).

#### *Plasmodium* phylogenetic analysis using nuclear genes

The nuclear markers were tested for amplification from fecal samples as well, however, they did not yield any positive results. Furthermore, to test the robustness of phylogenetic relationship depicted in Fig 2, we sequence two nuclear genes (*MSP-1*_*42*_ and *18s rRNA*) from the blood and tissue samples of these macaques. We could obtain six sequences of *MSP-1*_*42*_ gene (five *P*. *inui* and one *P*. *cynomolgi*) and nine sequences of *18s rRNA* (eight *P*. *inui*, one *P*. *cynomolgi*) from *M*. *radiata* derived *Plasmodium* samples (Table 2). However, we were unable to obtain sequences of these genes from rest of the samples, probably because of low parasitemia.

The *MSP-1*_*42*_ gene sequences obtained from six (five *P*. *inui* and one *P*. *cynomolgi*) Indian *Plasmodium* isolates were compared with the published sequences (S3 Table) available for simian malaria parasites. Contrary to *Cyt-b* gene sequences, *MSP-1*_*42*_ gene sequences were quite divergent among Indian *Plasmodium* species (0.015, Table 3) and the divergence between Indian and SEA *P*. *inui* sequences was quite high (0.036, Table 3). In case of *P*. *cynomolgi* the mean divergence between Indian and SEA sequences was 0.029 (Table 3). All the newly generated *Plasmodium MSP-1*_*42*_ gene sequences were deposited in GenBank sequence repository under accession numbers: *P inui* (MH974141, MH974143-MH974146), *P*. *cynomolgi* (MH974142).

A 429 bp fragment of *18s rRNA* gene was sequenced from two *Plasmodium* species (*P*. *inui* and *P*. *cynomolgi*) derived from Indian macaques. Similar to mitochondrial *Cyt-b* gene, eight Indian *P*. *inui* isolates of nuclear *18s rRNA* gene showed no genetic variation among them and these sequences exhibited very low divergence from other SEA *P*. *inui* sequences (0.004, Table 3). The single isolate of *P*. *cynomolgi* from India showed nucleotide substitutions at six sites when compared with the SEA *P*. *cynomolgi* isolates with the divergence value of 0.10 (Table 3).

**Fig 3:**
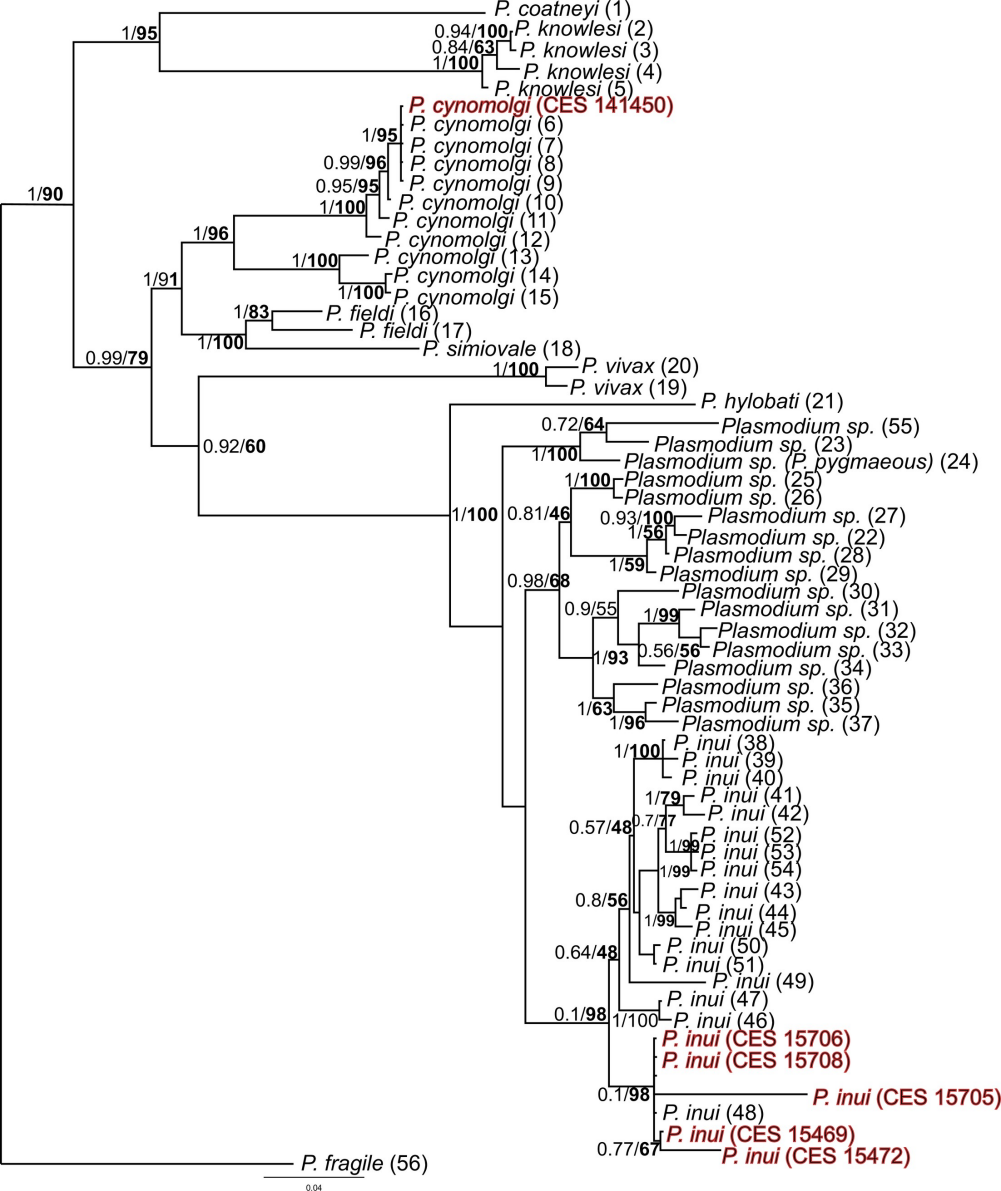
Phylogenetic tree of *Plasmodium* species based on nuclear genome *MSP-1*_*42*_ gene. Bayesian and ML methods yielded similar tree topologies and so only Bayesian tree is shown. The values above branches are posterior probabilities together with the bootstrap values (in bold) in percentage obtained for ML tree.

**Fig 4:**
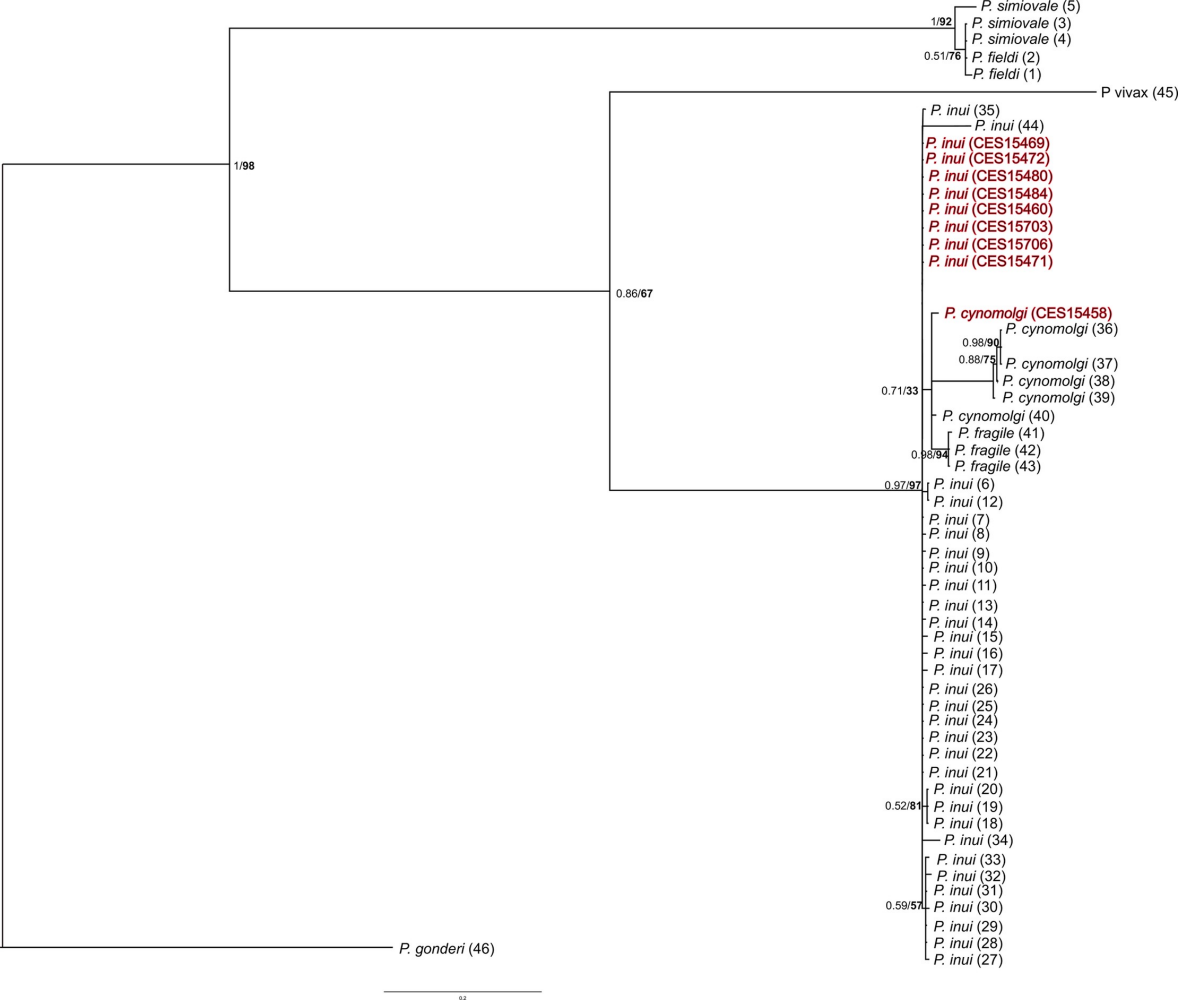
Phylogenetic tree of *Plasmodium* species based on nuclear genome *18s rRNA* gene. Bayesian and ML methods yielded similar tree topologies and so only Bayesian tree is shown. The values above branches are posterior probabilities together with the bootstrap values (in bold) in percentage obtained for ML tree.

Like in the mitochondrial tree the nuclear *MSP-*_*142*_ tree showed Indian *P*. *inui* and *P*. *cynomolgi* sequences were nested within their SEA counterparts (Fig 2 and Fig 3). In the *18s rRNA* gene tree *P*. *cynomolgi* from Indian and SEA along with *P*. *fragile* formed a clade (Fig 4). However, the position of *P*. *inui* was unresolved. Overall the *18s rRNA* gene tree was uninformative with respect to the position of *P*. *inui* and *P*. *cynomolgi* probably due to lack of sequences of other *Plasmodium* species. All the newly generated *Plasmodium 18s rRNA* gene sequences were deposited in GenBank sequence repository under accession numbers: *P*. *inui* (MH917236-MH917243), *P*. *cynomolgi* (MH917235).

### Phylogenetic analysis of host

Since we have sampled *M*. *radiata* from across the Southern India, covering most of its distribution area, we further wanted to determine if *M*. *radiata* exhibits any population structure. Since macaque groups are characterized by female philopatry [53], one might expect geographically structured mtDNA haplotype distribution. Nevertheless, “problem animals” are routinely translocated across India to curb monkey menace. This practice might in turn facilitate gene flow between previously isolated populations and aid in the spread of pathogens. Thus, we utilized the mitochondrial D-loop sequences to test if human mediated translocations might have altered the population structure of *M*. *radiata*. The Bayesian phylogenetic tree based on D-loop sequences is shown in S1 Fig. The tree topology does not support climate specific clustering of mtDNA clades, in that dry and wet zone individuals do not form distinct clades. Thus, the phylogeographic pattern suggests that macaques are freely moving across the dry and wet zones of the country. All the newly generated *Macaca radiata* D-loop sequences were deposited in GenBank sequence repository under accession numbers MH974147-MH974228.

### Host haplotype network and IBD model

A minimum spanning network of 42 *M*. *radiata* mtDNA haplotypes is depicted in S2 Fig. this network includes *M*. *radiata* haplotypes sampled across its distribution range in Southern India. The network analysis did not reveal geographical clustering of related haplotypes and in most cases very divergent and distantly related haplotypes were retrieved in a given population. Furthermore, in some cases identical haplotypes were derived from geographically distant populations. Thus, haplotype network also suggests a lack of population structure in this species. The results of IBD test was positive but very low (r = 0.298) suggesting week correlation between genetic and geographic distance, however this result was not significant (p = 0.09).

## Discussion

Indian macaques, chiefly *M*. *radiata*, have been known to harbour at least three *Plasmodium* species including *P*. *fragile*, *P*.*inui* and *P*. *cynomolgi*. However much of this work was done in the last century and since early 1980s there have been no studies on primate malaria in India. Here using *Plasmodium* sequences data isolated from the primate host and phylogenetic analyses we confirm the presence of these pathogens in *M*. *radiata*. Additionally, we report for the first time the presence of human malarial parasite, *P*. *falciparum*, in *M*. *mulatta* and *M*. *radiata*. This is also the first report of DNA sequence data obtained from *Plasmodium* species infecting wild populations of Indian macaques.

Overall the *P*. *inui* and *P*. *cynomolgi* sequences from India branch with their SEA counterparts in both nuclear and mitochondrial trees. Nevertheless, in some comparisons divergence between Indian and SEA sequences is higher than mean divergence among Indian sequences. However, the sample size is very limited. More studies need to be undertaken to determine if the Indian isolates represent a different species/subspecies. The *Plasmodium* endemic to Indian subcontinent, *P*. *fragile* branches with sequences from Sri Lanka and this species also exhibit high intraspecific variation. Among these three parasites, *P*. *cynomolgi* has been detected in several humans in the Nicobar Islands [54] and recently in a patient in Malaysia [55]. This raises the specter of yet another primate malarial parasite exhibiting zoonoses. Additionally, both *P*. *inui* and *P*. *cynomolgi* have been shown to infect humans in laboratory conditions [27, 56] and references therein. Thus, it is conceivable that these parasites have the potential to infect humans in mainland India.

### Presence of human malaria parasite in Indian macaques

The presence of human malarial parasite (*P*. *falciparum*) in *M*. *mulatta* and *M*. *radiata* was an unexpected finding but not surprising given such observations have been made before in other primates [15, 16]. However, this finding raises few questions, whether the parasite was able to infect the Indian macaques or it is because of the detection of pre-erythrocytic stage of the parasite. Since, the present study reports parasites from feces and the liver tissue only, one can assume that the detection was due to the pre-erythrocytic stage of the parasite. This is because asymptomatic pre-erythrocytic development of the mammalian malaria parasite occurs in liver, and the parasite might reach feces via bile. Thus, the PCR based method might pick up pre-erythrocytic stage of the parasites rather than erythrocytic infection in blood [57]. However, one cannot rule out the possibility that the parasite is able to infect the macaque RBCs, as the sample size of testing the blood samples was not enough to confirm. Given this scenario, there is an urgent need for screening more blood samples from Indian macaques using traditional methods of detecting blood stage parasites as well as molecular methods. Although, due to reasons like submicroscopic infections, morphological indistinguishable forms of *Plasmodium* and primate samples being opportunistic (as many primate species are endangered), the molecular based approaches are favored over the morphological data [4] (and references there in).

Previous studies have revealed that African primates (Apes) harbor at least six-host specific lineages representing distinct *Plasmodium* species within Laverania subgenus [17]. One of these lineages is closely related to human *P*. *falciparum* and thus referred as *P*. *falciparum* like malaria parasites. Since Ape specific *P*. *falciparum* were found to be more genetically diverse than human *P*. *falciparum*, it was hypothesized that human *P*. *falciparum* has evolved from *P*. *falciparum* like parasites found in Gorilla, by a single cross species transmission event [5]. These two *P*. *falciparum* lineages can be differentiated based on four SNPs from mitochondrial genome[5]. Interestingly, one of the recent studies from India found that few Indian human *P*. *falciparum* isolates shared one of the SNPs (out of the four above mentioned distinguishing SNPs) with *P*. *falciparum* like isolates, these samples also showed two novel SNPs. Moreover, they also found that Indian human *P*. *falciparum* bear high genetic and haplotype diversity. The authors conclude that Indian human *P*. *falciparum* might belong to the ancestral range of the species and it is likely that cross species transmission might have happened in India [58, 59]. Our finding of presence of *P*. *falciparum* in Indian macaques to some extent supports this hypothesis. However, based on the small fragment that we could sequence from macaques we are unable to determine if these sequences belong to human specific *P*. *falciparum* or *P*. *falciparum* like lineages isolated from non-human primates. Thus, for further understanding of *P*. *falciparum* origin and its host-switch mechanisms, more studies targeting Indian non-human primates are required.

### Geographic distribution of malaria parasites in Indian macaques

The distribution and spread of malarial parasites is tightly linked to the distributions of its vector and host. The only host known thus far for the primate specific malarial from Southern India is *M*. *radiata* which is distributed over much of peninsular India. However, most studies, including ours, have reported these parasites in *M*. *radiata* distributed in the high rainfall regions (wet zone) of Southwest India. According to [32] the distribution of macaque malaria appears to be governed by the distribution of the *Leucosphyrus* group of mosquitoes, which are largely confined to tropical evergreen forest. Evergreen forests in turn are restricted to Southwest India and the rest of the peninsula has semi-arid climate (here referred to as dry zone). The mosquito species *Anopheles elegans* has been implicated as the vector for these parasites in Southwest India. Nevertheless, in our study we also detected *P*. *inui* and *P*. *cynomolgi* from a captive population in Bangalore (PRC, IISc campus, Bangalore). The provenance of this captive population is not known but is most likely to have been locally sourced. Interestingly a new parasite named *P*. *osmaniae* was reported from free ranging monkeys from Hyderabad district in 1960 [60]. Later this species was reclassified as *P*. *inui* (see [27, 56]. Both Bangalore and Hyderabad are in the dry zone in central peninsular India. These observations suggest that *P*. *inui* and *P*. *cynomolgi* might be more widely distributed in Southern India than previously thought.

How do we explain the presence of these parasites in *M*. *radiata* from dry zone of peninsular India? There are two possible explanations. One plausible scenario is that the vector *A*. *elegans* has extended its range into the dry zone carrying the parasite with it and infecting host populations in the dry zone. Secondly infected members of host species might have dispersed from the wet zone of peninsular India to the dry zone and in these areas local *Anopheles* species (other than *A*. *elegans*) might have served as vectors. It is well known that under laboratory conditions many other species of *Anopheles* can transmit various macaque malarias [27, 56]. To explore these scenarios, we looked at the population structure and phylogeography of the host species, *M*. *radiata*.

### Phylogeography of *M*. *radiata*

*Macaca radiata* (Family Cercopithecidae) is a widely distributed and common species of macaque endemic to South India. It is distributed in wet and dry zones of peninsular India and is found in both forested areas as well as in human dominated landscapes. Like most other cercopithecines these macaques live in matrilineal troops wherein female offspring remain in their natal territory while male offspring disperse [61]. Such a social structure- termed female natal phylopatry results in geographical clustering on mtDNA haplotypes. This is because mtDNA is maternally inherited and therefore is restricted to a region due to female natal phylopatry. In many species of macaques such mtDNA structuring has been reported [62, 63].

However human mediated transport of macaques can disrupt this mtDNA population structure. Our study suggests that *M*. *radiata* does not exhibit any population structure in their mtDNA. For example, there is no segregation of wet and dry zone populations, they are interspersed in the phylogeny (S1 Fig). Furthermore, there is no geographical structuring of mtDNA haplotypes across the species’ range in that samples collected from 16 different locations are distributed across the network (S2 Fig). The IDB analysis also does not support a significant correlation between geographical and genetic distances. The lack of population structure in macaques we believe is largely due to human mediated dispersal. Across India “problem monkey” mainly from urban areas are trapped and released in their “native” forest habitat. These urban monkeys are usually unable to survive in forests as they have been acclimatized to foraging in urban environments. Often these translocated monkeys then move to nearby human habitations to forage. Such long-distance translocation results in the pattern seen in our analysis wherein individuals from distant locations have identical haplotype (Tumkur and Waynad) or individuals from the same location have very divergent haplotypes (Chickballapur).

Human mediated transport of macaques adds an additional level of complexity to tacking malaria. This issue has implications for both the spread of malaria in primates as well as humans. In the case of primate specific malaria such unnatural translocations would facilitates the wider distribution of these pathogens in their host species which in turn would provide more opportunities for zoonoses. Given that India is targeting complete malaria elimination by 2030 [64], our study recommends intensive spatial and temporal monitoring of primate malarial for a holistic approach to controlling of human malaria.

## Supporting information

S1 Fig Phylogenetic tree of *Macaca radiata* species based on mitochondrial D-loop region. Bayesian and ML methods yielded similar tree topologies and so only Bayesian tree is shown. The values above branches are posterior probabilities together with the bootstrap values (in bold) in percentage obtained for ML tree. Figure also depicts the wet zone (blue colored) and dry zone (brown colored) of Southwest India. The host fecal samples collected from the respective zones are colored accordingly in the phylogenetic tree.
(TIF)

S2 Fig Minimum spanning network of *M*. *radiata* D-loop mtDNA haplotypes from India. Branch lengths are proportional to number of nucleotide substitutions and node sizes are proportional to total haplotype frequencies.
(TIF)

S1 Table Details of fecal, blood and tissue samples collected from five different primate species of India. (DOCX)

S2 Table Details of published primate *Plasmodium* species *Cyt-b* gene sequences utilized for present phylogenetic reconstructions along with their natural hosts, geographic locations and accession numbers. (DOCX)

S3 Table Details of published primate *Plasmodium* species *MSP-1*_*42*_ gene sequences utilized for present phylogenetic reconstructions along with their natural hosts, geographic locations and accession numbers. (DOCX)

S4 Table Details of published primate *Plasmodium* species *18s rRNA* gene sequences utilized for present phylogenetic reconstructions along with their natural hosts, geographic locations and accession numbers. (DOCX)
