# Supplementary figures and images for: Reinvestigating the status of malaria parasite (Plasmodium sp.) in Indian non-human primates
Source: PLoS Negl Trop Dis. 2018 Dec 6;12(12):e0006801. doi: 10.1371/journal.pntd.0006801 (PMC6298686; doi:10.1371/journal.pntd.0006801)

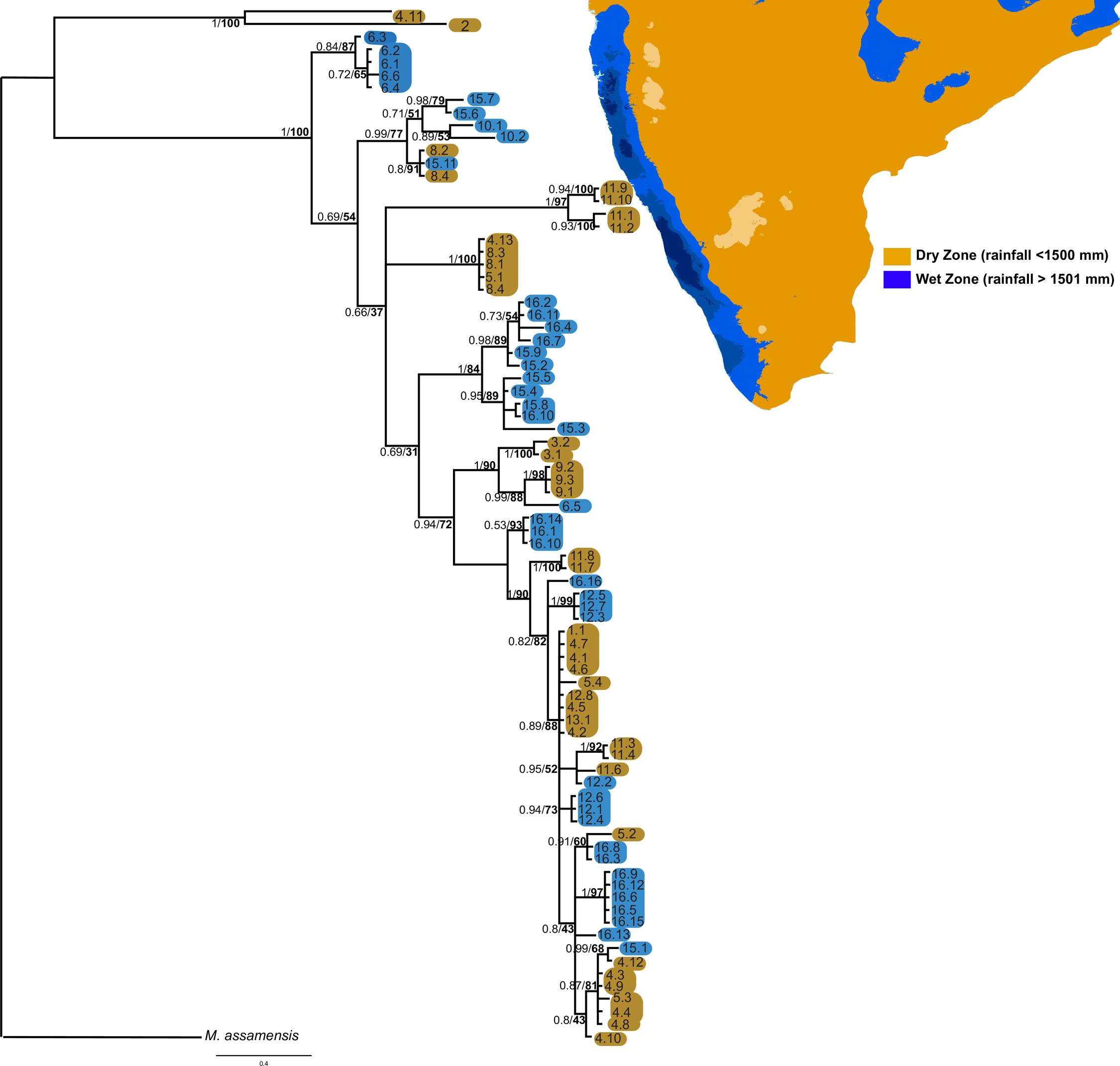

Supplement: S1 Fig — Bayesian and ML methods yielded similar tree topologies and so only Bayesian tree is shown. The values above branches are posterior probabilities together with the bootstrap values (in bold) in percentage obtained for ML tree. Figure also depicts the wet zone (blue colored) and dry zone (brown colored) of Southwest India. The host fecal samples collected from the respective zones are colored accordingly in the phylogenetic tree. (TIF) [file pntd.0006801.s001.tif]

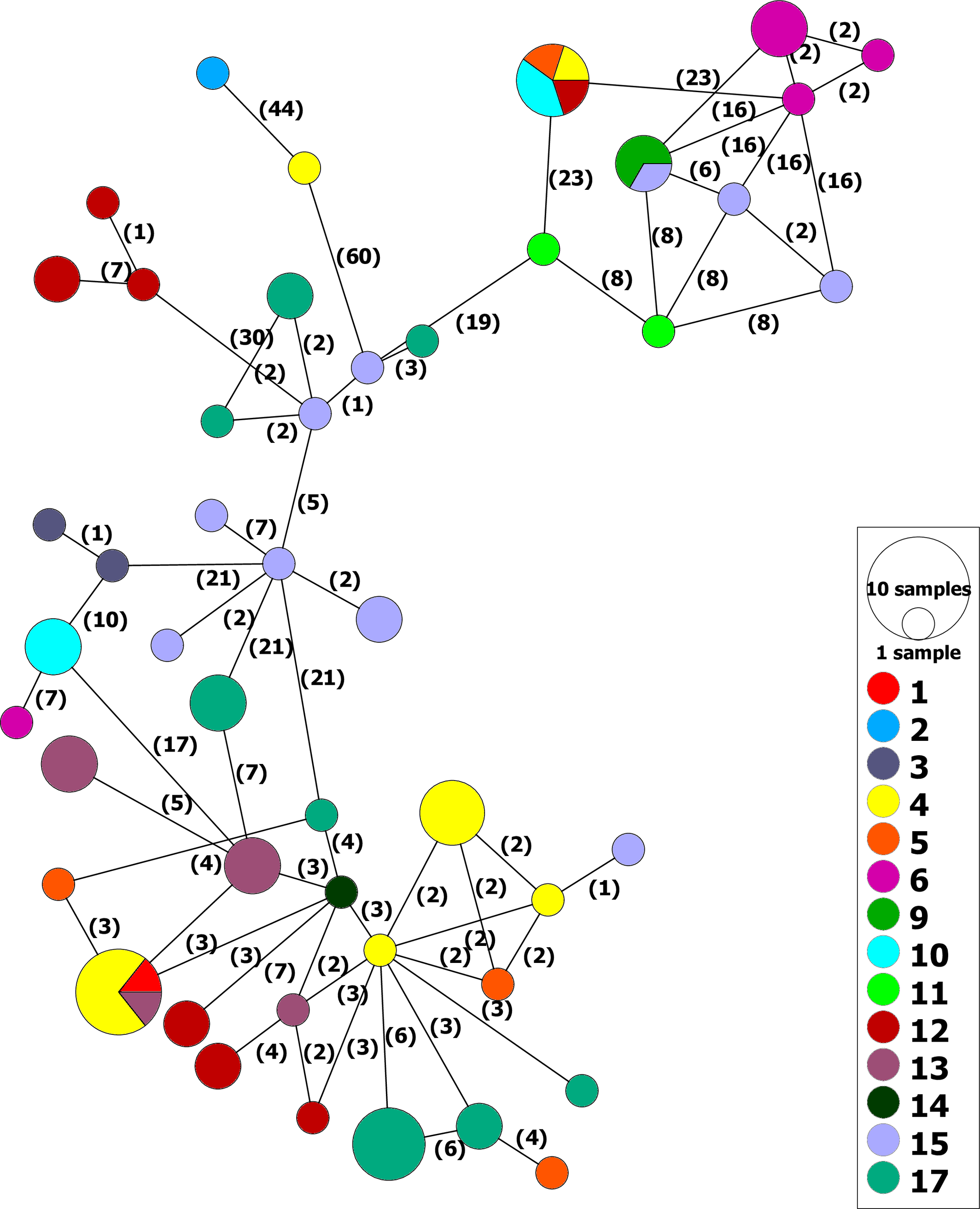

Supplement: S2 Fig — Branch lengths are proportional to number of nucleotide substitutions and node sizes are proportional to total haplotype frequencies. (TIF) [file pntd.0006801.s002.tif]
