# Supplementary material for: Reinvestigating the status of malaria parasite (Plasmodium sp.) in Indian non-human primates
Source: PLoS Negl Trop Dis. 2018 Dec 6;12(12):e0006801. doi: 10.1371/journal.pntd.0006801 (PMC6298686; doi:10.1371/journal.pntd.0006801)
Supplement: S1 Table — (DOCX) [file pntd.0006801.s003.docx]

S1Table: Collection details of fecal, blood and tissue samples collected from five different primate species of India.

| S. No. | Location | Species collected (Sample IDs) | | | | | total |
| --- | --- | --- | --- | --- | --- | --- | --- |
|  |  | *M. radiata* | *M. mulatta* | *M. fascicularis umbrosa* | *Symnopithecus hypoleucus* | *Macaca sinica* |  |
| 1 | Talakona guest house (Andhra) | 2 |  |  |  |  |  |
| 2 | Hatakeshwaram temple (Andhra) | 4 |  |  |  |  |  |
| 3 | Mehboobnagar (Telengana) | 3 |  |  |  |  |  |
| 4 | Chickbellapur (Karnataka) | 40 |  |  |  |  |  |
| 5 | Indian Institute of Sciences Bangalore (Karnataka) | 12 |  |  |  |  |  |
| 6 | Kudremukh, chickmangalore (Karnataka) | 33 |  |  | 5 | 6 |  |
| 7 | Mookambika, Udupi (karnataka) | 3 |  |  |  |  |  |
| 8 | Tumkur (karnataka) | 7 |  |  |  |  |  |
| 9 | Bellary (karnataka) | 5 |  |  | 6 |  |  |
| 10 | Ujire (karnataka) | 3 |  |  |  |  |  |
| 11 | Coimbatoor (T.N.) | 10 |  |  |  |  |  |
| 12 | Ooty (T.N.) | 31 |  |  |  |  |  |
| 13 | Salem (T.N.) | 8 |  |  |  |  |  |
| 14 | Sultan Bathery, Waynad (Kerala) | 27 |  |  |  |  |  |
| 15 | Kolpetta, Waynad, (Kerala) | 12 |  |  |  |  |  |
| 16 | Thrissur (Kerala) | 51 |  |  |  |  |  |
| 17 | Champaran (Bihar) |  | 2 |  |  |  |  |
| 18 | Delhi |  | 9 |  |  |  |  |
| 19 | Bahadurgarh (Haryana) |  | 10 |  |  |  |  |
| 20 | Nicobar Islands  (Andaman and Nicobar) |  |  | 57 |  |  |  |
| 21 | Lucknow (UP) |  | 3 |  |  |  |  |
| Total |  | 251 | 24 | 57 | 11 | 6 | 349 |
