# Supplementary material for: Reinvestigating the status of malaria parasite (Plasmodium sp.) in Indian non-human primates
Source: PLoS Negl Trop Dis. 2018 Dec 6;12(12):e0006801. doi: 10.1371/journal.pntd.0006801 (PMC6298686; doi:10.1371/journal.pntd.0006801)
Supplement: S2 Table — (DOCX) [file pntd.0006801.s004.docx]

S2 Table : Details of published primate species *Cyt-b* gene sequences utilized for present phylogenetic reconstructions along with their natural hosts, geographic locations and accession numbers.

| S. No. | Parasite | host | location | Accession Numbers |
| --- | --- | --- | --- | --- |
| 1 | *P. inui* | *Trachypithecus obscurus* | SEA | GQ355483 |
| 2 | *P. inui* | *Trachypithecus obscurus* | SEA | GQ355482 |
| 3 | *P. inui* | *M. nemestrina* | SEA | KJ569834 |
| 4 | *P. inui* | *M. cyclopis* | SEA | AB444118 |
| 5 | *P. inui* | *M. mulatta* | SEA | AB354572 |
| 6 | *P. inui* | *M. mulatta* | SEA | AB444116 |
| 7 | *P. inui* | *M. fascicularis* | SEA | AB444115 |
| 8 | *P. inui* | *Trachypithecus obscurus* | SEA | AB444109 |
| 9 | *P. inui* | *M. nemestrina* | SEA | AB444112 |
| 10 | *P. inui* | *M. fascicularis* | Malaysia | AB444119 |
| 11 | *P. inui* | *Anopheles leucophyrus* | West Malaysia | AB444114 |
| 12 | *P. inui* | *M. nemestrina* | West Malaysia | AB444113 |
| 13 | *P. inui* | *Macaca species* | - | AB444111 |
| 14 | *P. inui* | *Presbytis obscurus* | West Malaysia | AB444117 |
| 15 | *P. inui* | *M. nigra* | Indonesia | AB444120 |
| 16 | *P. inui* | *M. fascicularis* |  | AF069617 |
| 17 | *P. inui* | Wild macaques | Thailand | EU400398 |
| 18 | *P. inui* | Wild macaques | Thailand | EU400410 |
| 19 | *P. inui* | *Macaca species* | West Malaysia | AB444110 |
| 20 | *P. hylobatid* | *Hylobates molock* | Indonesia | AB354573 |
| 21 | *Plasmodium species* | *Pongo pygmaeus* | Indonesia | JQ308531 |
| 22 | *Plasmodium species* | *Pongo pygmaeus morio* | Malaysia | KJ569827 |
| 23 | *Plasmodium species* | *Pongo pygmaeus morio* | Malaysia | KJ569814 |
| 24 | *Plasmodium species* | *Pongo pygmaeus morio* | Malaysia | KJ569804 |
| 25 | *Plasmodium simiovale* | *M. sinica* | Sri Lanka | AB434920 |
| 26 | *Plasmodium fieldi* | *M. nemestrina* | West Malaysia | AB354574 |
| 27 | *Plasmodium fieldi* | *Anopheles balabacensis introlatus* | West Malaysia | AB444132 |
| 28 | *Plasmodium fragile* | *M. sinica* | Sri Lanka | AY722799 |
| 29 | *Plasmodium fragile* | *M. sinica* | Sri Lanka | AB444136 |
| 30 | *Plasmodium fragile* | *M. radiata* | India | AB444135 |
| 31 | *Plasmodium vivax* | *Pan troglodytes* | Uganda | GQ355481 |
| 32 | *Plasmodium vivax* | *Homo sapiens* | El Salvador | AY598140 |
| 33 | *Plasmodium vivax* | *Pan troglodytes* | Republic of Congo | GQ355480 |
| 34 | *Plasmodium gonderi* | *Cercocebus species* | Africa | AY800111 |
| 35 | *Plasmodium sp.* | Mandrill | Africa | AY800112 |
| 36 | *Plasmodium coatneyi* | Asian wild macaques | SEA | AB354575 |
| 37 | *Plasmodium coatneyi* | Asian wild macaques | Southern Thailand | EU400407 |
| 38 | *Plasmodium knowlesi* | SEA Wild macaque | Malaysia | NC_007232 |
| 39 | *Plasmodium species_ M. nemestrina* | *M. nemestrina* | Malaysia | KJ569854 |
| 40 | *Plasmodium cynomolgi* | *M. nemestrina* | West Malaysia | AB444129 |
| 41 | *Plasmodium cynomolgi* | *M. nemestrina* | West Malaysia | AB444126 |
| 42 | *Plasmodium cynomolgi* | *M. fascicularis* | Malaysia | KJ569868 |
| 43 | *Plasmodium cynomolgi* | *M. fascicularis* | Malaysia | KJ569867 |
| 44 | *Plasmodium cynomolgi* | *M. fascicularis* | Malaysia | KJ569866 |
| 45 | *Plasmodium cynomolgi* | *M. nemestrina* | Malaysia | KJ569865 |
| 46 | *Plasmodium cynomolgi* | Asian old-world monkeys | Srilanka | AB444125 |
| 47 | *Plasmodium cynomolgi* | Asian old-world monkeys | Srilanka | AF069616 |
| 48 | *Plasmodium cynomolgi* | *M. nemestrina* | West Malaysia | AB444128 |
| 49 | *Plasmodium cynomolgi* | *M. fascicularis* | Malaysia | AB444127 |
| 50 | *Plasmodium cynomolgi* | Asian old-world monkeys | SEA | AB444123 |
| 51 | *Plasmodium cynomolgi* | Asian old-world monkeys | SEA | AB434919 |
| 52 | *Plasmodium cynomolgi* | Asian old-world monkeys | SEA | AY800108 |
| 53 | *Plasmodium cynomolgi* | *M. speciosa* | Assam | AB444131 |
| 54 | *Plasmodium cynomolgi* | Asian old-world monkeys | SEA | AB444130 |
| 55 | *Plasmodium cynomolgi* | *M. fascicularis* | Combodia | AB444124 |
| 56 | *Plasmodium cynomolgi* | Asian old-world monkeys | SEA | AB444122 |
| 57 | *Plasmodium cynomolgi* | Asian old-world monkeys | SEA | AB444121 |
