# Supplementary material for: Reinvestigating the status of malaria parasite (Plasmodium sp.) in Indian non-human primates
Source: PLoS Negl Trop Dis. 2018 Dec 6;12(12):e0006801. doi: 10.1371/journal.pntd.0006801 (PMC6298686; doi:10.1371/journal.pntd.0006801)
Supplement: S3 Table — (DOCX) [file pntd.0006801.s005.docx]

S3 Table: Details of published primate species *MSP-1_42_* gene sequences utilized for present phylogenetic reconstructions along with their natural hosts, geographic locations and accession numbers.

| S. No. | Parasite | host | location | Accession Number |
| --- | --- | --- | --- | --- |
| 1 | *Plasmodium coatneyi* | Wild macaque | SEA | AB266180 |
| 2 | *Plasmodium knowlesi* | SEA Wild macaque | SEA | AB266184 |
| 3 | *Plasmodium knowlesi* | SEA Wild macaque | SEA | XM_0022585446 |
| 4 | *Plasmodium knowlesi* | SEA Wild macaque | Thailand | DQ220743 |
| 5 | *Plasmodium knowlesi* | SEA Wild macaque | SEA | DQ907701 |
| 6 | *Plasmodium cynomolgi* | SEA Wild macaque | SEA | DQ907682 |
| 7 | *Plasmodium cynomolgi* | SEA Wild macaque | SEA | DQ907680 |
| 8 | *Plasmodium cynomolgi* | SEA Wild macaque | SEA | AY869723 |
| 9 | *Plasmodium cynomolgi* | SEA Wild macaque | SEA | DQ907681 |
| 10 | *Plasmodium cynomolgi* | SEA Wild macaque | SEA | DQ907683 |
| 11 | *Plasmodium cynomolgi* | SEA Wild macaque | SEA | DQ907679 |
| 12 | *Plasmodium cynomolgi (Ceylonensis strain)* | SEA Wild macaque | SEA | DQ907684 |
| 13 | *Plasmodium cynomolgi* | SEA Wild macaque | SEA | DQ907678 |
| 14 | *Plasmodium cynomolgi* | SEA Wild macaque | SEA | DQ907676 |
| 15 | *Plasmodium cynomolgi* | SEA Wild macaque | SEA | DQ907677 |
| 16 | *Plasmodium fieldi* | *Macaca nemestrina* | West Malaysia | AB444064 |
| 17 | *Plasmodium fieldi* | *Macaca nemestrina* | West Malaysia | AB444065 |
| 18 | *Plasmodium simiovale* | SEA Wild macaque | SEA | AB266185 |
| 19 | *Plasmodium vivax* | Human | China | JX993755 |
| 20 | *Plasmodium vivax* | Human | Central America | XM_001614792 |
| 21 | *Plasmodium hylobatid* | *Hylobates* | SEA | DQ907700 |
| 22 | *Plasmodium sp.* | *Pongo pygmaeus morio* | Malaysia | KJ569911 |
| 23 | *Plasmodium sp.* | *Pongo pygmaeus morio* | Malaysia | KJ569916 |
| 24 | *Plasmodium sp.* | *Pongo pygmaeus morio* | Malaysia | KJ569917 |
| 25 | *Plasmodium sp.* | *Pongo pygmaeus morio* | Malaysia | KJ569914 |
| 26 | *Plasmodium sp.* | *Pongo pygmaeus morio* | Malaysia | KJ569913 |
| 27 | *Plasmodium sp.* | *Pongo pygmaeus morio* | Malaysia | KJ569909 |
| 28 | *Plasmodium sp.* | *Pongo pygmaeus morio* | Malaysia | KJ569910 |
| 29 | *Plasmodium sp.* | *Pongo pygmaeus morio* | Malaysia | KJ569912 |
| 30 | *Plasmodium sp.* | *Pongo pygmaeus morio* | Malaysia | KJ569904 |
| 31 | *Plasmodium sp.* | *Pongo pygmaeus morio* | Malaysia | KJ569901 |
| 32 | *Plasmodium sp.* | *Pongo pygmaeus morio* | Malaysia | KJ569903 |
| 33 | *Plasmodium sp.* | *Pongo pygmaeus morio* | Malaysia | KJ569902 |
| 34 | *Plasmodium sp.* | *Pongo pygmaeus morio* | Malaysia | KJ569906 |
| 35 | *Plasmodium sp.* | *Pongo pygmaeus morio* | Malaysia | KJ569907 |
| 36 | *Plasmodium sp.* | *Pongo pygmaeus morio* | Malaysia | KJ569905 |
| 37 | *Plasmodium sp.* | *Pongo pygmaeus morio* | Malaysia | KJ569908 |
| 38 | *Plasmodium inui* | *Macaca nemestrina* | Malaysia | KJ569800 |
| 39 | *Plasmodium inui* | *Macaca nemestrina* | Malaysia | KJ569801 |
| 40 | *Plasmodium inui* | *Macaca nemestrina* | Malaysia | KJ569802 |
| 41 | *Plasmodium inui* | *Macaca nemestrina* | Malaysia | KJ569803 |
| 42 | *Plasmodium inui* | *Macaca nemestrina* | Phillipines | DQ907690 |
| 43 | *Plasmodium inui* | SEA Wild macaque | Malaysia | DQ907691 |
| 44 | *Plasmodium inui* | SEA Wild macaque | Malaysia | DQ907692 |
| 45 | *Plasmodium inui* | SEA Wild macaque | Malaysia | DQ907693 |
| 46 | *Plasmodium inui* | SEA Wild macaque | Malaysia | DQ907694 |
| 47 | *Plasmodium inui* | SEA Wild macaque | Malaysia | DQ907695 |
| 48 | *Plasmodium inui* | SEA Wild macaque | Malaysia | DQ907696 |
| 49 | *Plasmodium inui* | SEA Wild macaque | Malaysia | DQ907699 |
| 50 | *Plasmodium inui* | SEA Wild macaque | Malaysia | DQ907685 |
| 51 | *Plasmodium inui* | SEA Wild macaque | Malaysia | DQ907686 |
| 52 | *Plasmodium inui* | SEA Wild macaque | Malaysia | DQ907687 |
| 53 | *Plasmodium inui* | SEA Wild macaque | Taiwan | DQ907688 |
| 54 | *Plasmodium inui* | SEA Wild macaque | Malaysia | DQ907689 |
| 55 | *Plasmodium sp.* | *Pongo pygmaeus morio* | Malaysia | KJ569915 |
| 56 | *Plasmodium fragile* | SEA Wild macaque | Nilgiri strain | DQ907702 |
