# Supplementary material for: Reinvestigating the status of malaria parasite (Plasmodium sp.) in Indian non-human primates
Source: PLoS Negl Trop Dis. 2018 Dec 6;12(12):e0006801. doi: 10.1371/journal.pntd.0006801 (PMC6298686; doi:10.1371/journal.pntd.0006801)
Supplement: S4 Table — (DOCX) [file pntd.0006801.s006.docx]

S4 Table: Details of published primate species *18s rRNA* gene sequences utilized for present phylogenetic reconstructions along with their natural hosts, geographic locations and accession numbers.

| S. No. | Plasmodium species | Host species | location | Accession number |
| --- | --- | --- | --- | --- |
| 1 | *Plasmodium fieldi* | Asian old-world monkeys | Malaysia | FJ619102 |
| 2 | *Plasmodium fieldi* | *M. fascicularis* | Malaysia | KC662444 |
| 3 | *Plasmodium simiovale* | Asian old-world monkeys | SEA | AB287286 |
| 4 | *Plasmodium simiovale* | Asian old-world monkeys | SEA | AB287287 |
| 5 | *Plasmodium simiovale* | Asian old-world monkeys | SEA | AB287285 |
| 6 | *Plasmodium inui* | Asian wild macaques | Thailand | EU400384 |
| 7 | *Plasmodium inui* | Asian wild macaques | Thailand | EU400385 |
| 8 | *Plasmodium inui* | Asian wild macaques | Thailand | EU400386 |
| 9 | *Plasmodium inui* | Asian wild macaques | Thailand | EU400387 |
| 10 | *Plasmodium inui* | Asian wild macaques | Thailand | EU400388 |
| 11 | *Plasmodium inui* | Asian wild macaques | Thailand | EU400389 |
| 12 | *Plasmodium inui* | Asian wild macaques | Thailand | EU400390 |
| 13 | *Plasmodium inui* | Asian wild macaques | Thailand | EU400391 |
| 14 | *Plasmodium inui* | Asian wild macaques | Thailand | EU400392 |
| 15 | *Plasmodium inui* | Asian wild macaques | Thailand | EU400395 |
| 16 | *Plasmodium inui* | Asian wild macaques | Thailand | EU400396 |
| 17 | *Plasmodium inui* | Asian wild macaques | Thailand | EU400397 |
| 18 | *Plasmodium inui* | *M. fascicularis* | Malaysia | FJ619076 |
| 19 | *Plasmodium inui* | *M. fascicularis* | Malaysia | FJ619078 |
| 20 | *Plasmodium inui* | *M. fascicularis* | Malaysia | FJ619079 |
| 21 | *Plasmodium inui* | *M. fascicularis* | Malaysia | FJ619081 |
| 22 | *Plasmodium inui* | *M. fascicularis* | Malaysia | FJ619082 |
| 23 | *Plasmodium inui* | *M. fascicularis* | Malaysia | FJ619085 |
| 24 | *Plasmodium inui* | *M. fascicularis* | Malaysia | FJ619093 |
| 25 | *Plasmodium inui* | *M. fascicularis* | Malaysia | FJ619095 |
| 26 | *Plasmodium inui* | *M. fascicularis* | Malaysia | FJ619104 |
| 27 | *Plasmodium inui* | *M. cyclopis* | Taiwan | FN256224 |
| 28 | *Plasmodium inui* | *M. cyclopis* | Taiwan | FN256225 |
| 29 | *Plasmodium inui* | *M. cyclopis* | Taiwan | FN256226 |
| 30 | *Plasmodium inui* | *M. cyclopis* | Taiwan | FN256227 |
| 31 | *Plasmodium inui* | *M. cyclopis* | Taiwan | FN256228 |
| 32 | *Plasmodium inui* | *M. cyclopis* | Taiwan | FN256229 |
| 33 | *Plasmodium inui* | *M. cyclopis* | Taiwan | FN256230 |
| 34 | *Plasmodium inui* | Asian wild macaques | SEA | PIU72541 |
| 35 | *Plasmodium inui* | *M. fascicularis* | South China | HM032051 |
| 36 | *Plasmodium cynomolgi* | Asian wild macaques | Malaysia | KC581783 |
| 37 | *Plasmodium cynomolgi* | Asian wild macaques | Malaysia | KC581784 |
| 38 | *Plasmodium cynomolgi* | Asian wild macaques | Malaysia | KC662439 |
| 39 | *Plasmodium cynomolgi* | Asian wild macaques | SEA | AB287288 |
| 40 | *Plasmodium cynomolgi* | *M. fascicularis* | Malaysia | FJ619084 |
| 41 | *Plasmodium fragile* | Asian wild macaques | SEA | AB287273 |
| 42 | *Plasmodium fragile* | Asian wild macaques | SEA | AB287274 |
| 43 | *Plasmodium fragile* | Asian wild macaques | SEA | M61722 |
| 44 | *Plasmodium inui* | Asian wild macaques | China | XR606809 |
| 45 | *Plasmodium vivax* | Human | China | KT991325 |
| 46 | *Plasmodium gonderi* | *Cercocebus* species | Africa | AY579416 |
